# Iron‐Locked Hydr(oxy)oxide Catalysts via Ion‐Compensatory Reconstruction Boost Large‐Current‐Density Water Oxidation

**DOI:** 10.1002/advs.202300717

**Published:** 2023-04-07

**Authors:** Jiao Liu, Wei Du, Siying Guo, Jing Pan, Jingguo Hu, Xiaoyong Xu

**Affiliations:** ^1^ College of Physics Science and Technology Yangzhou University Yangzhou 225002 China

**Keywords:** electrochemical reconstruction, large‐current‐density stability, nickel‐iron hydr(oxy)oxides, oxygen evolution reaction

## Abstract

Nickel‐iron based hydr(oxy)oxides have been well recognized as one of the best oxygen‐evolving catalysts in alkaline water electrolysis. A crucial problem, however, is that iron leakage during prolonged operation would lead to the oxygen evolution reaction (OER) deactivation over time, especially under large current densities. Here, the NiFe‐based Prussian blue analogue (PBA) is designed as a structure‐flexible precursor for navigating an electrochemical self‐reconstruction (ECSR) with Fe cation compensation to fabricate a highly active hydr(oxy)oxide (NiFeO*_x_* H*_y_*) catalyst stabilized with Ni—Fe synergic active sites. The generated NiFeO*_x_* H*_y_* catalyst exhibits the low overpotentials of 302 and 313 mV required to afford large current densities of 500 and 1000 mA cm^−2^, respectively. Moreover, its robust stability over 500 h at 500 mA cm^−2^ stands out among the NiFe‐based OER catalysts reported previously. Various in/ex situ studies indicate that the Fe fixation by dynamic reconstruction process can reinforce the Fe‐activated effect on the OER amenable to the industrial‐level large current conditions against the Fe leakage. This work opens up a feasible strategy to design highly active and durable catalysts via thermodynamically self‐adaptive reconstruction engineering.

## Graphical abstract

The authors showcase an effective strategy to anchor Fe‐activated reactive sites in NiFeO*_x_* H*_y_* matrices by ion compensatory reconstruction over structure‐flexible precursors and thus design highly active and robust hydr(oxy)oxide catalysts competent in industrial water electrolysis.

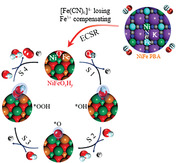

## 1 Introduction

The water electrolysis is an eco‐friendly and promising strategy to generate green hydrogen (H_2_) fuel.^[^ ^1^, ^2^, ^3^ ^]^ In this process, the oxygen evolution reaction (OER) at the anode is more kinetically sluggish compared with the hydrogen evolution reaction (HER) at the cathode, dominating the majority of power dissipation.^[^ ^4^, ^5^ ^]^ Therefore, the development of high‐performance and low‐cost catalysts to reduce OER overpotential and minimize energy consumption is highly desired for industrial applications.^[^ ^6^, ^7^, ^8^, ^9^, ^10^ ^]^ With considerable endeavor on transition metal‐based catalysts, the nickel‐iron hydr(oxy)oxides (NiFeO*_x_* H*_y_*) emerge among the most excellent earth‐abundant catalysts for alkaline OER^[^ ^11^, ^12^, ^13^, ^14^, ^15^ ^]^ and some even outperform precious RuO_2_/IrO_2_ benchmark catalysts. Moreover, recent in situ/operando studies have found that the diverse NiFe‐based catalysts, including alloys,^[^ ^16^, ^17^ ^]^ sulfides,^[^ ^18^, ^19^ ^]^ nitrides^[^ ^20^ ^]^ and oxides,^[^ ^21^, ^22^ ^]^ underwent dynamic reconstruction under OER conditions and generated similar NiFeO*_x_* H*_y_* phases as their really active species.^[^ ^16^, ^17^, ^18^, ^19^, ^20^, ^21^, ^22^, ^23^, ^24^, ^25^ ^]^ Along this line, the NiFeO*_x_* H*_y_* have attracted increasing research interest as an anodic catalyst alternative promising in industrial water electrolysis.^[^ ^26^, ^27^, ^28^, ^29^, ^30^ ^]^

Although the catalytic mechanism remains controversial for the NiFeO*_x_* H*_y_* catalyst,^[^ ^31^, ^32^, ^33^, ^34^, ^35^ ^]^ it is undoubted that the interaction between Ni and Fe is vital to stimulate the high OER activity and the O‐bridged Ni—Fe moieties can be referred to synergic reaction centers.^[^ ^11^, ^36^ ^]^ Burke et al. revealed the OER activity trend for 3d‐metal hydr(oxy)oxides (MO*_x_* H*_y_*) follows the order Fe > Co > Ni.^[^ ^37^ ^]^ The recent density functional theory (DFT) calculations also disclosed that the Fe site endows with fast OER kinetics but which only works in eligible conducting hosts like the NiOOH matrices while fails in insulating FeOOH.^[^ ^38^ ^]^ In addition, Chung et al. recently examined the stability of MOOH (M = Ni, Co, Fe) under OER conditions and found that the FeOOH was easily soluble with significantly higher dissolution rate than that of NiOOH.^[^ ^39^ ^]^ Taking both activity and stability into consideration, the conducting NiOOH substance with high durability despite relatively poor activity can serve as a reliable host for anchoring Fe to cultivate magical Fe effect, and thus the Ni—O—Fe motifs responsible for high OER performance dominate the superiority of NiFeO*_x_* H*_y_* catalysts.

Recent studies on the long‐term stability of NiFeO*_x_* H*_y_* catalysts have shown that the Fe leakage was a common issue resulting in the activity degradation with prolonged OER,^[^ ^40^, ^41^, ^42^, ^43^ ^]^ especially under large current densities. So, how to stabilize the Fe‐relevant segments in the NiFeO*_x_* H*_y_* catalyst becomes a significant challenge.^[^ ^44^ ^]^ More recently, the surface metal dissolution/redeposition dynamics over the NiFeO*_x_* H*_y_* catalyst during the OER process has been suggested to understand and intervene with the activity change over time.^[^ ^45^, ^46^, ^47^, ^48^ ^]^ Accordingly, two new concepts associated with dynamic Fe sites of “reversible phase segregation” and “dynamically stable active sites” have been proposed to regenerate or sustain the Fe‐activated phases for prolonging a catalyst's lifetime.^[^ ^39^, ^48^ ^]^ The Co and Fe mixed metal salts in the electrolyte have applied for in situ dynamic generation of active species to handle the catalyst's leakage problem during a long‐term continuous OER process.^[^ ^49^ ^]^ These pioneering advances open up some questions, for example, how the interface ion‐exchange dynamics is controlled and whether it is adapted to an industrial‐level large current density condition. Our group recently uncovered the chameleon‐like reconstruction dynamics toward highly active species self‐adaptive to the electrocatalytic environment.^[^ ^50^, ^51^ ^]^ These studies motivated us to consider whether magic Fe sites can be assembled or even reinforced via dissolution/deposition dynamics by electrochemical self‐reconstruction (ECSR) over advisable precursors. It may be an avenue to induce the tightness and adequacy in hosting incorporated Fe for robust NiFeO*_x_* H*_y_* generation.

Herein, we deliberately design a flexible NiFe Prussian blue analogue (PBA, K_2_Ni[Fe(CN)_6_]·nH_2_O) with the cyanide (CN) groups‐bridged bimetallic NiFe open frameworks to execute an online monitoring over reconstruction dynamics under continuously strong anodic polarization with high current densities. Together with the introduction of ferric nitrate in the electrolyte, we demonstrate the feasibility of Fe locked into the NiO*_x_* H*_y_* host through the complete reconstruction process with dissolution–deposition dynamics. Accordingly, a Fe‐stabilized NiFeO*_x_* H*_y_* catalyst was in situ constructed after sufficiently thermodynamic relaxation, which exhibits both superior OER activity and stability in alkaline media. The continuous running at 500 mA cm^−2^ steady for 500 h supports the NiFeO*_x_* H*_y_* catalyst stepping toward practical applications. Further an intentional aging test at an extremely high current density of 1000 mA cm^−2^ shows a gradual activity decline related to the slow Fe leakage, and then the Fe refixation via ion‐compensatory reconstruction can revivify the degraded activity. Therefore, we believe that the dominant active centers are the Ni—O—Fe motifs formed by the Fe incorporation into the NiO*_x_* H*_y_* host, which can be stabilized and regenerated by Fe‐feeding reconstruction process. This work showcases an effective strategy to anchor Fe‐activated reactive sites in MO*_x_* H*_y_* hosts by ion compensatory reconstruction over structure‐flexible precursors for designing highly active and robust hydr(oxy)oxide catalysts.

## 2 Results and Discussion

### 2.1 Synthesis and Characterization of NiFe PBA Precursor

**Figure 1:**
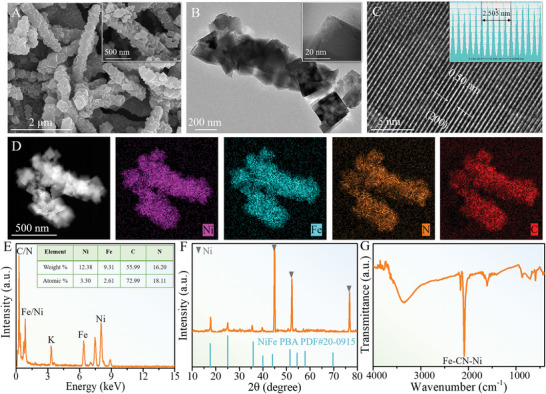
Morphology and microstructure of NiFe PBA precursor. A) SEM image with a magnified inset, B) TEM image with a magnified inset, and C) HRTEM image of NiFe PBA. D) TEM‐EDX elemental mappings, E) EDX spectrum with atomic ratios (inset), F) XRD pattern, and G) FTIR spectrum of NiFe PBA.

The 1D NiFe PBA nanoarrays (NAs) self‐supported on nickel foam (NF) were obtained through a two‐step process, that is, a growth of the NiMoO_4_ nanorods in hydrothermal method followed by an ion exchange by soaking in K_3_[Fe(CN)_6_] aqueous solution (see the synthesis details in the Experimental Section, Figure S1, Supporting Information). From scanning electron microscopy (SEM) images, the regular NiMoO_4_ nanorods, with the smooth surface and uniform size of ≈300 nm in diameter, successfully template the generation of 1D NiFe PBA NAs (Figure S2, Supporting Information). **Figure** **1**A shows the SEM morphology of as‐synthesized NiFe PBA NAs, in which the 1D units consist of a large number of nanocubes (NCs) connected together and inherit an average diameter of around 300 nm similar to the precursor nanorods (the inset in Figure 1A). The transmission electron microscopy (TEM) images further display the pillar‐like structure constructed from uniform cubic nanocrystals of around 200 nm in size with smooth surfaces and sharp edges (Figure 1B and the inset). The high‐resolution TEM (HRTEM) image over individual NC (Figure 1C) shows the bright lattice fringes with *d*‐spacing of 0.50 nm that can be indexed to the (200) plane of NiFe PBA, disclosing its highly crystalline nature with the regular atom arrangement, as further evidenced by the atomic intensity profile along [200] orientation (the inset). The corresponding fast Fourier transform (FFT) and selected area electron diffraction (SAED) spectra (Figure S3, Supporting Information) together demonstrate the formation of well‐defined NiFe PBA with the high crystallinity. The energy‐dispersive X‐ray (EDX) elemental mapping images (Figure 1D) visualize the homogeneous distribution of Ni, Fe, C and N elements in the NiFe PBA, and the EDX spectroscopy (Figure 1E) reveals the atomic percentages of metallic Ni and Fe close to each other. The X‐ray diffraction (XRD) pattern (Figure 1F) displays that the diffraction peaks are well assigned to the NiFe PBA crystals (PDF no. 20–0915) besides the peaks from NF substrate. Notice that there are no other diffraction peaks including that of the NiMoO_4_ template, indicating the complete transformation into the PBA by an ion‐exchange process (Figure S4, Supporting Information). In addition, the Fourier transform infrared (FTIR) spectrum (Figure 1G) directly exhibits the bonds of Fe‐CN‐Ni at the range between 2000 and 2200 cm^−1^, suggesting the formation of the NiFe PBA with metal‐organic open framework.

### 2.2 Controlled Reconstruction of NiFe PBA Precursor

Bimetallic PBA materials have been emerged as an intriguing OER (pre)catalysts in the photo/electrochemical energy fields.^[^ ^52^, ^53^, ^54^ ^]^ Electrochemically‐induced reconstruction over NiFe PBA has been also discovered by Zhang et al. to identify the newly generated NiO*_x_* H*_y_* layer after Fe losing as an actual contributor to OER activity.^[^ ^55^ ^]^ Recently, Yu et al. reported that the generation of unconventional CN vacancies in the NiFe PBA via ionized nitrogen plasma treatment effectively suppresses the loss of Fe element, enabling the formation of mixed metal NiFeO*_x_* H*_y_* layer with enhanced OER activity.^[^ ^44^ ^]^ These studies lead us to speculate that the design of NiFe PBA precatalyst with resoluble [Fe(CN)_6_]^4−^ groups followed by the strategical ECSR in an intentionally adding Fe content electrolyte could in situ create the desirable NiFeO*_x_* H*_y_* species via the dissolution and deposition mechanism. Therefore, we here employed in situ Raman and inductively coupled plasma mass spectrometry (ICP‐MS) methods to investigate the dynamic ECSR and whereby designed the robust Fe‐stabilized NiFeO*_x_* H*_y_* catalyst competent for industry‐level high‐current‐density OER.

**Figure 2:**
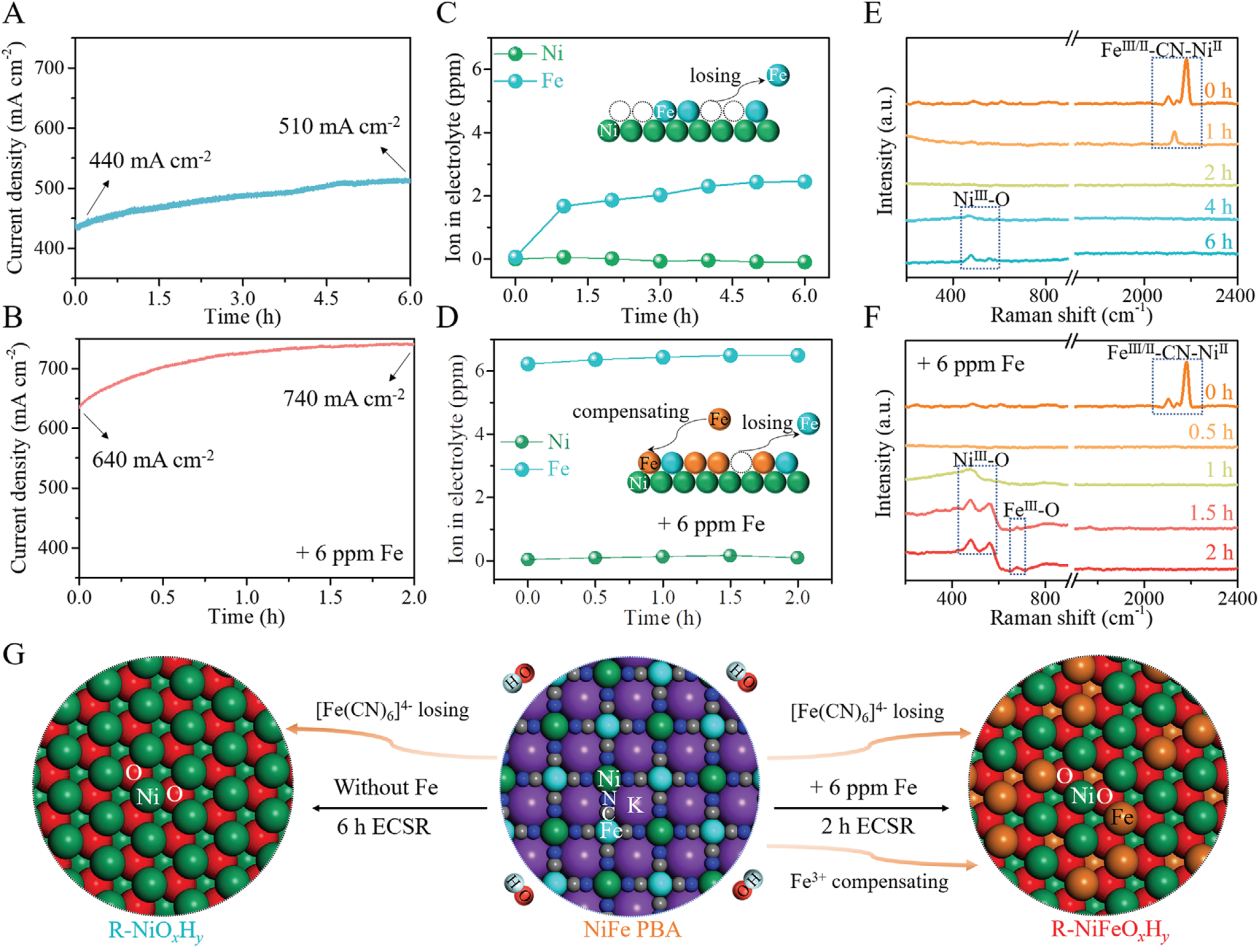
In situ studies on the ECSR of NiFe PBA. A,B) CA *j* −*t* curves of NiFe PBA at applied 1.2 V potential in 1 m KOH with and without Fe addition (6 ppm). C,D) In situ ICP‐MS plots with the schematic for Fe losing/compensating dynamics (insets), and E,F) Raman spectra of NiFe PBA during CA measurements at applied 1.2 V voltage in 1 m KOH with and without Fe addition. G) Schematic illustration of reconstruction dynamics on NiFe PBA toward different R‐NiO*_x_* H*_y_* and R‐NiFeO*_x_* H*_y_* products upon Fe‐cation compensation.

Continuously anodic polarization in chronoamperometry (CA, *j* − *t*) was applied on the NiFe PBA at constant 1.2 V potential to drive the ECSR at room temperature using a standard three‐electrode setup in 1.0 m KOH electrolytes with and without Fe addition, respectively. Note that the commercial reagent‐grade KOH aqueous solution contains the trace Fe content of around 0.06 ppm and the maximum content of Fe‐ion saturated KOH solution with the addition of ferric nitrate reaches ≈6.22 ppm. The former and the latter are used as two contrast electrolytes labeled as “without/with Fe addition”, respectively, for easy discrimination. In 1 m KOH electrolyte without Fe addition, the NiFe PBA shows the gradually increased current density from 440 to 510 mA cm^−2^ within 6 h under anodic potential at 1.2 V (**Figure** **2**A), indicating an activity enhancement accompanied by the ECSR occurrence. Surprisingly, the Fe adding to the electrolyte significantly facilitates the activity growth with current density increasing from 640 to 740 mA cm^−2^ at equilibrium within 2 h under the same test conditions, which implies the discrepant reconstruction course toward different products. The ECSR dependence on the Fe presence in electrolyte is further demonstrated by the line sweep voltammetry (LSV) curves before and after the CA activation (Figure S5, Supporting Information). The Ni and Fe cation concentrations in the electrolytes were tracked in situ by using the ICP‐MS method (Table S1, Supporting Information). As shown in Figure 2B, an increase of the Fe content in 1 KOH electrolyte reveals the Fe leakage from the NiFe PBA during the ECSR process, especially in the first hour. Whereas in the Fe‐added electrolyte, the Fe leakage is effectively restrained with a negligible Fe‐content change (Figure 2D). No detectable Ni leakage is found for both the two activation processes. The Fe survival ratios in final catalysts after activation equilibrium are extremely different, ≈0% and ≈63% for two cases without and with Fe addition, respectively, basing on the X‐ray photoelectron spectroscopy (XPS) analysis on catalysts (Table S2, Supporting Information). In situ Raman tracking on the NiFe PBA precatalysts during two CA processes in different electrolytes further captures the microstructural evolution signals (Figure 2E,F). Initial Raman peaks at 2179 and 2127 cm^−1^ correspond to the vibrations of Fe^III^‐CN‐Ni^II^ and Fe^II^‐CN‐Ni^II^ in NiFe PBA, respectively.^[^ ^56^ ^]^ Under the anodic polarization, the intensity of Fe^II/III^‐CN‐Ni^II^ bands is quickly reduced and almost undetectable after 2 h either with or without Fe compensation in electrolytes, indicating the complete dissolution of [Fe(CN)_6_]^4−^ during ECSR process. Interestingly, with the anodic polarization over time, new Raman peaks emerge and noticeably differ between two cases without and with Fe adding. In the pristine KOH electrolyte, the two peaks appear at 560 and 480 cm^−1^, characteristic of the *A*_1g_ stretching and *E*_g_ bending modes in Ni^III^—O bonds,^[^ ^57^ ^]^ respectively, reveals the generation of *γ*‐NiOOH. In contrast, two extra signals at 423 and 680 cm^−1^ associated with the Fe—O vibration in hydr(oxy)oxide matrices are observed when Fe adding in electrolyte,^[^ ^58^, ^59^ ^]^ indicating that the Fe can incorporate into NiOOH during the ECSR. Moreover, the Fe incorporation induces an increase of $I_{A_{1g}}$/$I_{E_{g}}$ ratio (Figure S6, Supporting Information), which suggests the lattice disorder in NiOOH with Fe incorporation.^[^ ^34^ ^]^ In addition, ex‐situ FTIR spectrum on the reconstructed catalyst with Fe compensation exhibits three bands at 637, 872 and 3346 cm^−1^ (Figure S7, Supporting Information), ascribed to the Ni—O—H and Fe—O—H bending modes as well as the —OH stretching mode,^[^ ^60^, ^61^ ^]^ respectively, which further identifies the resultant NiFeO*_x_* H*_y_* species. Notice that the Fe—O—H bending mode at 872 cm^−1^ is not discerned on the reconstructed catalyst in pristine KOH electrolyte due to the irreversible Fe loss, consistent with in situ Raman analysis. Basing on above studies, we become clear on the reconstruction dynamics upon Fe‐cation compensation, which includes the [Fe(CN)_6_]^4−^ dissolution and simultaneous formation of NiOOH with Fe incorporation, rendering the bimetallic NiFeO*_x_* H*_y_* product as a finally stable OER catalyst, as schematically illustrated in Figure 2G. Whereas, no Fe‐compensatory reconstruction leads to the monometallic NiO*_x_* H*_y_* product after [Fe(CN)_6_]^4−^ losing, in absence of desired Fe effect on OER upgrade.

### 2.3 Microstructural Characterizations for Reconstructed Catalysts

**Figure 3:**
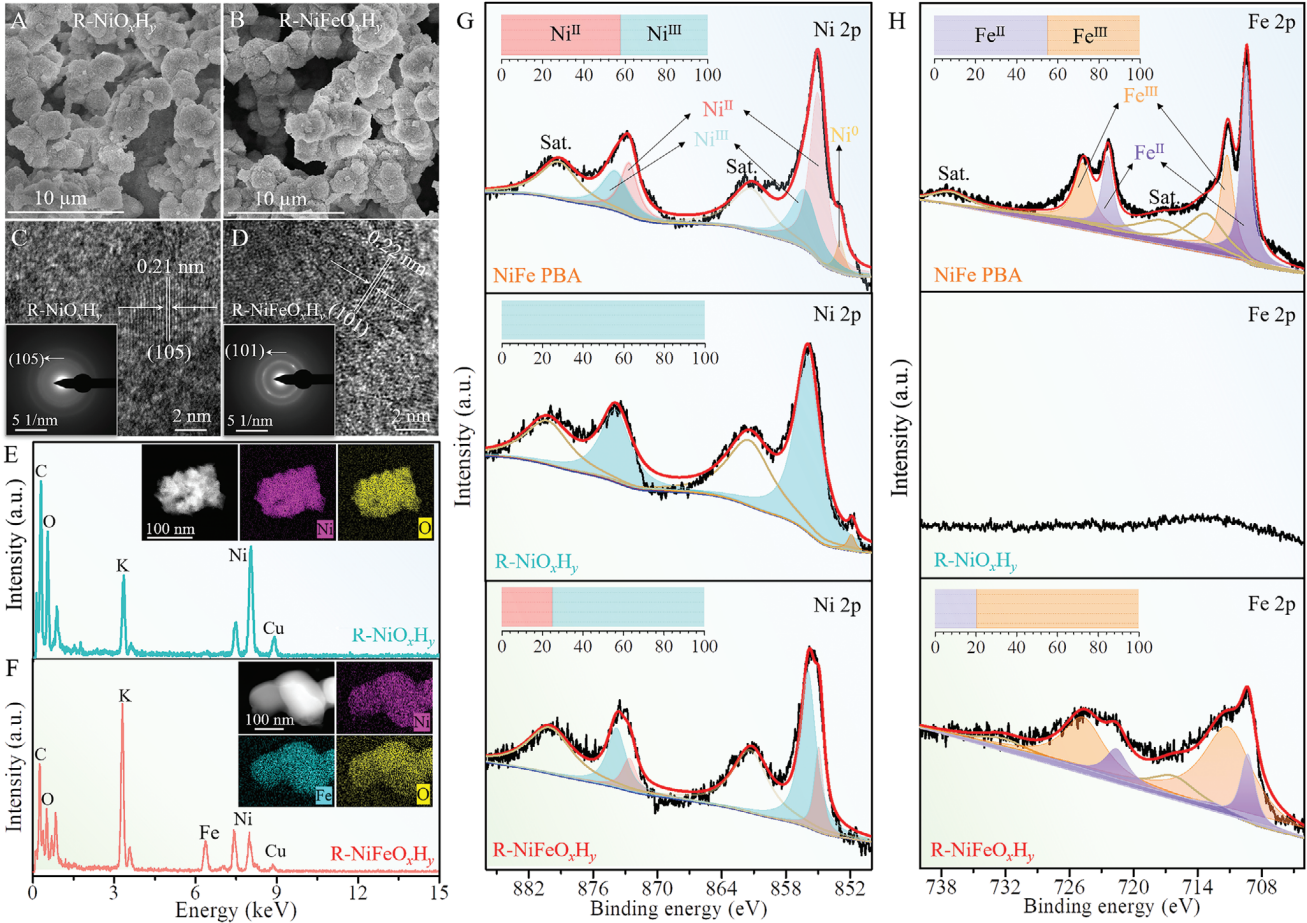
Morphology and microstructure of reconstructed products. A,B) SEM images, C,D) HRTEM images with SAED patterns (inset), as well as E,F) EDX spectra with elemental mappings (insets) for R‐NiO*_x_* H*_y_* and R‐NiFeO*_x_* H*_y_* catalysts. High‐resolution XPS spectra of G) Ni‐2p and H) Fe‐2p cores for NiFe PBA, R‐NiO*_x_* H*_y_*, and R‐NiFeO*_x_* H*_y_*.

Different products via above two ECSR processes are labeled as R‐NiO*_x_* H*_y_* and R‐NiFeO*_x_* H*_y_*, respectively, and first compared in microstructures by the SEM, TEM and EDX characterizations. The SEM imaging (**Figures** **3**A and 3B) shows that both R‐NiO*_x_* H*_y_* and R‐NiFeO*_x_* H*_y_* catalysts have the 3D porous network structure connected by NC unites, which obviously benefits from the 1D array template of NiFe PBA precursor. The changes in morphology to flocculent shapes can be observed for both R‐NiO*_x_* H*_y_* and R‐NiFeO*_x_* H*_y_* under close‐up TEM imaging (Figure S8, Supporting Information), compared to well‐shaped NCs of pristine NiFe PBA. The flocculent species reconstructed during OER usually correspond to the derivation of layer‐structured hydr(oxy)oxides.^[^ ^47^, ^50^ ^]^ The high‐resolution TEM (HRTEM) images (Figures 3C and 3D) disclose their low crystalline texture, in which some short‐range ordered lattice domains of NiOOH (105) plane can be discerned for R‐NiO*_x_* H*_y_*, and that of NiFeOOH (101) plane for R‐NiFeO*_x_* H*_y_*, respectively, consistent with their selected area electron diffraction (SAED) patterns (insets). Note that such a quasi‐amorphous characteristic has been found to be common for reconstructed active catalysts,^[^ ^62^, ^63^ ^]^ which endows with abundant crystalline‐amorphous boundaries rich in coordination‐unsaturated active sites accessible to catalysis. More importantly, no detectable Fe element survives in R‐NiO*_x_* H*_y_* under the EDX analysis (Figure 3E), while R‐NiFeO*_x_* H*_y_* contains mixed Ni—Fe elemental composition with uniform distributions (Figure 3F). These post‐reconstruction characterizations further verified the regulation of Fe‐cation compensation over the reconstruction dynamics on the NiFe PBA precursor toward different products. Noting that the Ni—Fe mixed hydr(oxy)oxide catalyst here derives by the ECSR with high‐current‐density OER conditions, presumably featured by Fe‐activated site stabilization immune to harsh thermodynamics.

We further performed the XPS measurements to probe the metal valence states in different products after two contrast ECSR processes. For the NiFe PBA, the XPS spectrum of Ni 2p (Figure 3G) displays two spin‐orbit peaks of Ni 2p_1/2_ and Ni 2p_3/2_ with binding energies at 855.0 and 873.0 eV, respectively, and two shakeup satellites at around 879.5 and 861.3 eV, besides a small signal peak from metallic Ni^0^ in NF substrate. The Ni 2p_1/2_ and Ni 2p_3/2_ bands can be resolved into four peaks located at 855.0 and 872.6 eV for Ni^II^, as well as 856.3 and 874.0 eV for Ni^III^, respectively.^[^ ^59^ ^]^ Likewise, the Fe 2p_1/2_ and Fe 2p_3/2_ bands in the Fe 2p spectrum (Figure 3H) can also be deconvoluted into four peaks at 709.4 and 722.5 eV for Fe^II^, as well as 711.2 and 724.9 eV for Fe^III^, respectively.^[^ ^64^ ^]^ The result indicates that both Ni and Fe elements exist in multivalent states, with the proportional columns of different valence states shown in the insets (Table S3, Supporting Information). After the ECSR into the R‐NiO*_x_* H*_y_*, the dominant Ni^III^ peaks within the convolution suggest that most of Ni atoms oxidize into trivalent states due to the NiOOH formation, while the Fe signals almost disappear after subjection to complete Fe leaching. For the ECSR with Fe‐cation compensation, the generated R‐NiFeO*_x_* H*_y_* is enriched with high valence metal atoms (Ni^3+^/Fe^3+^) in metal‐mixed hydr(oxy)oxide framework. Overall, XPS results are consistent with the above proposed reconstruction dynamics including Fe dissolution/incorporation and metal oxidation toward the hydr(oxy)oxides, in which whether Fe could be incorporated depends on Fe‐cation compensation.

In addition, the R‐NiO*_x_* H*_y_* and R‐NiFeO*_x_* H*_y_* exhibit their O 1s spectra with higher intensity than NiFe PBA precursor (Figure S9, Supporting Information). The deconvoluted peaks at 529.7, 531.6 and 533.0 eV can be assigned to the divalent metal‐coordinated oxygen (M^II^—O, e.g., in oxide or hydroxide), trivalent metal‐coordinated oxygen (M^III^—O, e.g., in hydroxyoxide), and surface absorbed water, respectively.^[^ ^47^ ^]^ The M^III^—O components significantly increase in both R‐NiO*_x_* H*_y_* and R‐NiFeO*_x_* H*_y_*, because of metal oxidizing to hydroxyoxides. The XPS signal of N 1s completely disappears in both NiO*_x_* H*_y_* and NiFeO*_x_* H*_y_* (Figure S10, Supporting Information), which further confirms the leakage of [Fe(CN)_6_]^4−^ groups during ECSR processes.

### 2.4 Electrochemical Studies for Reconstructed Catalysts

**Figure 4:**
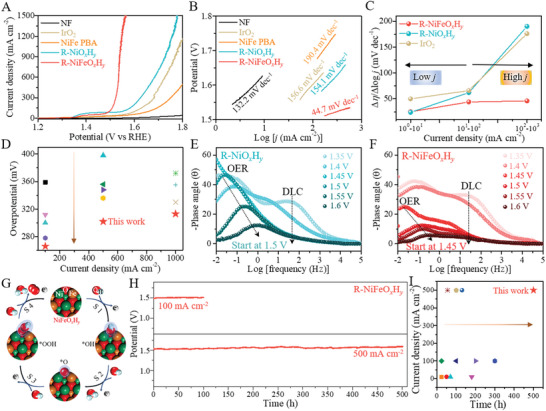
Electrocatalytic OER performance and mechanism. A) LSV polarization curves and B) Tafel slops of the NiFe PBA, R‐NiO*_x_* H*_y_* and R‐NiFeO*_x_* H*_y_*, with the bare NF and commercial IrO_2_ as two references. C) Ratio of Δ*η*/Δlog*j* of the R‐NiO*_x_* H*_y_*, R‐NiFeO*_x_* H*_y_*, and IrO_2_ in various current density sections. D) Overpotential comparison at various current densities for the R‐NiFeO*_x_* H*_y_* and other advanced catalysts reported in literatures. E,F) Bode phase diagrams of in situ EIS spectra for the R‐NiO*_x_* H*_y_* and R‐NiFeO*_x_* H*_y_*. G) Schematic diagram of four‐step OER mechanism at Ni—Fe synergic sites of the R‐NiFeO*_x_* H*_y_*. H) CP stability tests for the R‐NiFeO*_x_* H*_y_* at various current densities. I) Durability comparison at various current densities for the R‐NiFeO*_x_* H*_y_* and other advanced catalysts reported in literatures.

The OER activities of relevant catalysts were examined by the LSV measured at a scan rate of 5 mV s^−1^ in 1 m KOH solution with a standard three‐electrode system, and all potentials were recorded through the standard *i*R‐correction and calibration versus the reversible hydrogen electrode (RHE). As shown in **Figure** **4**A, the R‐NiFeO*_x_* H*_y_* is the best polarization performing, and its current density in LSV raises faster compared to the R‐NiO*_x_* H*_y_*, NiFe PBA, bare NF, and commercial IrO_2_ benchmark. For instance, the overpotentials needed for the R‐NiFeO*_x_* H*_y_* to render typical current densities of 10, 100, 500 and 1000 mA cm^2^ are 227, 272, 302 and 313 mV, respectively, which are considerably lower than those of the R‐NiO*_x_* H*_y_*, NiFe PBA and IrO_2_ (Figure S11, Supporting Information). Correspondingly, the R‐NiFeO*_x_* H*_y_* exhibits the smallest Tafel slope as low as 44.7 mV/dec (Figure 4B), indicative of highly intrinsic OER kinetics. To evaluate the activity at large current densities, a descriptor of Δ*η*/Δlog*j* was estimated in different current density (*j*) ranges to illustrate how much overpotential (*η*) is required for current density growth (Figure 4C).^[^ ^65^ ^]^ By comparison, only the R‐NiFeO*_x_* H*_y_* can maintain a small ratio of Δ*η*/Δlog*j* less than 50 mV/dec as the current density increases among the above contrast catalysts, while the others exhibit the overpotential surges for feeding the current growth especially when beyond 100 mA cm^−2^. Electrochemical impedance spectroscopy (EIS) was employed to assess the charge transfer resistance (*R*_ct_) at the catalyst/reactant interfaces. The Nyquist plots (Figure S12, Supporting Information) show that the *R*_ct_ of R‐NiFeO*_x_* H*_y_* is only 1.8 Ohms, much smaller than those of the R‐NiO*_x_* H*_y_* (≈3 Ohms) and NiFe PBA (≈14 Ohms), indicating preferable OER dynamics on the R‐NiFeO*_x_* H*_y_* surface. These results suggest the positive effect of incorporated Fe on OER activation in line with the findings of most literatures,^[^ ^17^, ^47^ ^]^ and conclude that our R‐NiFeO*_x_* H*_y_* has extraordinary OER activity even under industrial‐level large current densities, which surpasses most of NiFe‐based catalysts reported so far (Figure 4D and Table S4, Supporting Information).

To understand the role of Fe incorporation in enhancing OER activity, we further studied the electrochemical properties for the R‐NiFeO*_x_* H*_y_* and R‐NiO*_x_* H*_y_* in terms of the cycle voltammetry (CV) and in situ EIS. According to the non‐Faraday CV curves, the electrochemically active surface areas (ECSA) were calculated (Figure S13, Supporting Information), where the R‐NiFeO*_x_* H*_y_* has a slightly higher ECSA value than that of the R‐NiO*_x_* H*_y_*. Even so, when the LSV curves were normalized to the ECSA (Figure S14, Supporting Information), the R‐NiFeO*_x_* H*_y_* still shows significantly superior polarization capacity to the R‐NiO*_x_* H*_y_*, uncovering the Fe‐relevant action on enhanced intrinsic OER activity. In situ EIS measurements were conducted to gain insight into the electrical charge kinetics at catalyst/electrolyte interface from the double‐layer capacitance (DLC) to catalytic OER transfer. In the Bode phase diagrams of the R‐NiO*_x_* H*_y_* (Figure 4E) and R‐NiFeO*_x_* H*_y_* (Figure 4F), there are two indicator peaks at the high‐frequency and low‐frequency region regions, which are ascribed to the DLC and OER behaviors, respectively.^[^ ^18^ ^]^ By comparison, the R‐NiFeO*_x_* H*_y_* displays larger DLC behavior at the same applied potentials, and stronger polarization with oxhydryl accumulation induces greater catalytic driving force. With the potential increasing, the decrease in the low‐frequency phase angle occurs earlier for the R‐NiFeO*_x_* H*_y_* (at 1.45 V) in comparison with the R‐NiO*_x_* H*_y_* (at 1.50 V), and the corresponding Nyquist semicircle of the R‐NiFeO*_x_* H*_y_* reduces more rapidly (Figure S15, Supporting Information). These results further demonstrate that the R‐NiFeO*_x_* H*_y_* is easier to be polarized to initiate OER, consistent with the above conclusion of Fe‐enhanced intrinsic activity.

We then investigated the pseudocapacitive properties during the CV processes to compare the Ni redox features for the R‐NiFeO*_x_* H*_y_* and R‐NiO*_x_* H*_y_* (Figure S16, Supporting Information). The increment of Ni valency prior to the OER stage can be found in the pseudocapacitive region (1.35–1.48 V), which corresponds to the deprotonation process on Ni sites. Whereby, we note the muting effect of Fe component on Ni oxidation in the R‐NiFeO*_x_* H*_y_*, in agreement with earlier findings on the anodic shifts of Ni oxidizing waves by Fe dopants,^[^ ^17^, ^66^ ^]^ implying a different deprotonation mechanism involving with Fe sites for mixed Ni—Fe catalysts. Recent theoretical calculations have indicated that Fe is more flexible than Ni for redox response and the bimetallic Ni—Fe center favors O* intermediates to lower the energy barrier of the pivotal deprotonation step from OH* to O*.^[^ ^11^ ^]^ Thus, we conceivably assume the O‐bridged Ni—Fe centers as synergetic reaction sites to proceed proton/electron transfer for evolving OER intermediates on the R‐NiFeO*_x_* H*_y_* surface (Figure 4G). When the deprotonation step (S1) starts at the bridged OH to the Ni and Fe sites, the electrooxidation of the Ni^II^ cations could be alleviated by preferential polarizing at neighboring Fe sites with less electronegativity, and moreover the polarized Fe^III^ sites could favorably trigger the subsequent O* and OOH* transitions into OER steps (S2–S4). Hence the synergy from Fe sites lowers the energy barriers to smooth reactant‐to‐intermediate and intermediate‐to‐product transformations, in this way achieving nearly steady‐state cascade reaction from reactant to product. In contrast, when electrically polarizing the single Ni sites for the R‐NiO*_x_* H*_y_* to reach an energy level accessible to chemical OER, the anodic potential gap (Δ*V*) between Ni oxidation and OER onset is noticeably larger than that for the R‐NiFeO*_x_* H*_y_* (Figure S17, Supporting Information). The OER onset far from intermediates evolving suggests the formidable energy barrier from intermediates to product transition on single Ni—Ni sites. Thus, we believe that the role of Fe is likely to not only mediate the deprotonation mode but also facilitate OER kinetics, thereby leading to an enhanced OER activity.

In addition to activity, another critical character of stability should also be considered especially under large current densities when evaluating OER catalysts for practical applications. Long‐term durability tests for the R‐NiFeO*_x_* H*_y_* catalyst was conducted in multistep chronopotentiometry (CP) method with various current densities in 1 m KOH. As shown in Figure 4H, there is negligible overpotential loss at either 100 or 500 mA cm^−2^ over time, indicating an excellent stability for the R‐NiFeO*_x_* H*_y_* under alkaline OER conditions. The recorded video shows intense oxygen and hydrogen bubbling over the R‐NiFeO*_x_* H*_y_* working electrode and graphite rod counter electrode (Video S1, Supporting Information). In particular, the record of the R‐NiFeO*_x_* H*_y_* steady at 500 mA cm^−2^ over 500 h outperforms the stability test levels reported for most Ni—Fe based catalysts (Figure 4I and Table S5, Supporting Information). We further examined the activity and stability of the R‐NiFeO*_x_* H*_y_* working in purified KOH electrolyte (Figure S18, Supporting Information), where no significant difference from that in unpurified electrolyte excludes the influence of trace Fe impurities in 1 KOH solution. We thus conclude that the robust performance of the R‐NiFeO*_x_* H*_y_* profits from its own fixed Fe sites rather than the dynamically exchanged Fe sites via dissolution/redeposition process, which is likely inadequate to support the intense interfacial mass/charge transfer at large current densities. We then deliberately carried out the stability breaking test at 1000 mA cm^−2^ (Figure S19, Supporting Information), the overpotential shows a gradual increase for 24 h and meanwhile the in situ ICP‐MS measurement discovers the simultaneous Fe dissolution that accounts for the OER deactivation. A series of microstructural characterizations for post‐test catalyst on SEM, TEM, EDX, Raman, and XPS techniques (Figure S20, Supporting Information) further confirm that the catalyst aging arises from Fe losing rather than physical detachment or other structural changes. Amazingly, when the treatment of Fe‐cation compensatory reconstruction was repeated on aging R‐NiFeO*_x_* H*_y_* catalyst, similar OER activity as original that can be obtained again (Figure S21, Supporting Information). Furthermore, the Raman and XPS spectra (Figure S22, Supporting Information) disclose the repair of crucial Fe sites in Fe^III^—O coordination after repeated ECSR. These results suggest that the fastness of Fe component substantially determines the catalyst performance and its stability upper limit of running current density. Our R‐NiFeO*_x_* H*_y_* catalyst fixed with Ni—Fe synergic centers by ion‐compensatory reconstruction can withstand large current densities up to 500 mA cm^−2^, in which the Fe‐hosted stabilization is amenable to industrial‐level working intensity due to an experience of thermodynamic relaxation under harsh conditions. Moreover, the self‐recovery function can project ion‐compensatory reconstruction as an engaging strategy to produce self‐healing catalytic electrodes, which can increase cost‐effectiveness in practical applications.

## 3 Conclusion

In summary, we reported an ion‐compensatory reconstruction strategy on composition‐soluble NiFe PBA precursor to in situ generate highly active and robust NiFeO*_x_* H*_y_* catalyst reinforced with Ni—Fe synergic active sites for alkaline water oxidation. The resultant NiFeO*_x_* H*_y_* catalyst show an overpotential as low as 303 mV at 500 mA cm^−2^ and an extraordinary stability over 500 h, which are among the best reported for Ni—Fe based OER catalysts. Such remarkable OER performance is ascribed to the fixation of Fe‐activated sites during the dynamic reconstruction process with thermodynamic relaxation under large current densities. Various online and offline techniques capture the Fe survival and leakage in controlled catalysts, demonstrating the valuable Fe effect triggered in adjacent Ni—Fe active centers on catalytic activity and stability. Our findings pave a new avenue for designing high‐performance OER catalysts and stimulate further interests on in situ ECSR engineering to develop advanced catalysts in other electrochemical fields.

## Conflict of Interest

The authors declare no conflict of interest.

## Supporting information

Supporting Information

Supplemental Video 1

## Data Availability

The data that support the findings of this study are available from the corresponding author upon reasonable request.
