# Supplementary material for: Iron‐Locked Hydr(oxy)oxide Catalysts via Ion‐Compensatory Reconstruction Boost Large‐Current‐Density Water Oxidation
Source: Adv Sci (Weinh). 2023 Apr 7;10(16):2300717. doi: 10.1002/advs.202300717 (PMC10238203; doi:10.1002/advs.202300717)
Supplement: Supplementary file 1 — Supporting Information [file ADVS-10-2300717-s001.pdf]

Supporting Information

Iron-Locked Hydr(oxy)oxide Catalysts via Ion-Compensatory Reconstruction Boost Large-Current-Density Water Oxidation

*Jiao Liu, Wei Du, Siying Guo, Jing Pan, Jingguo Hu, and Xiaoyong Xu**

† Electronic supplementary information (ESI) available: Experimental Details and Additional Characterizations.

1. Experimental Section

Synthesis of NiFe PBA NAs precursor. The one-dimensional (1D) nanoarrays (NAs) of NiFe Prussian blue analogue (PBA) were synthesized on nickel foam (NF) through a two-step process. Briefly, $NiMoO_4$ nanorods (NRs) were fabricated by a hydrothermal reaction, followed by an ion exchange reaction in $K_3[Fe(CN)_6]$ solution to obtain NiFe PBA with 1D-templeted structure. First, a piece of NF with the area of 1×3 cm^2 and thickness of 1 mm was washed successively in 1 M HCl aqueous solution, deionized (DI) water and ethanol using ultrasonication. Second, 0.01 mmol of $Na_2MoO_4 \cdot 2H_2O$ and 0.04 mmol of $Ni(NO_3)_2 \cdot 6H_2O$ were mixed together with 50 mL of DI water under magnetic stirring for 10 min and formed a homogenous solution. Third, this mixture with one piece of NF was transferred into a 25 mL Teflon-lined autoclave, which was sealed and heated at 150 °C for 6 h reaction, afterwards the NF was taken out and washed with ethanol and DI water several times before being fully dried at 60 °C for 24 h. Finally, the as-synthesized NF-supported $NiMoO_4$ NRs were immersed into 8 mg L^{-1} $K_3[Fe(CN)_6]$ solution at 90 °C for 2 h to obtain NiFe PBA NAs.

Fabrication of IrO_2-loaded electrodes. To fabricate the IrO_2 electrode for comparison, 4.8 mg of commercial IrO_2 was added into a dispersion solution containing 300 μL ethanol, 150 μL deionized water and 30 μL Nafion. The mixed sol was treated with ultrasonication for 30 min, and then coated onto cleaned NF substrate and dried overnight in air.

Material Characterization. X-ray diffraction (XRD) was performed on a Shimadzu XRD-7000 diffractometer with Cu Kα radiation (λ = 1.54 Å) at a scanning rate of 2° min^{-1} in the 2θ range of 10–80°. Scanning electron microscopy (SEM) was taken on a Hitachi S-4800II instrument at 5 kV acceleration voltage. Transmission electron microscopy (TEM) was carried out on a Tecnai F30 instrument at 100 kV acceleration

voltage, with energy dispersed X-ray (EDX) analysis. Fourier transform infrared spectroscopy (FTIR) was recorded on a Varian 670-IR spectrometer with the spectral range of 400–4000 cm^{-1}. In situ Raman spectroscopic experiments were performed using a self-built system assembled by a three-electrode water splitting cell coupled with RPB4 spectrometer with 532 nm laser excitation source. X-ray photoelectron spectroscopy (XPS) was performed on an ESCALAB250Xi spectrometer using Al Kα as an excitation source at a power of 150 W, and all binding energies were referenced to the C 1s peak at 284.8 eV. Inductively coupled plasma-mass spectrometry (ICP-MS) was taken on Elan DRC-e (PerkinElmer).

Electrochemical measurements. All electrochemical tests were conducted on a CHI660E system in a standard three-electrode setup at room temperature in 1 M KOH electrolyte. The self-supported catalysts on NF substrates were directly used as the working electrode, while a graphite rod and a Hg/HgO electrode were applied as the counter and reference electrodes, respectively. Continuously anodic self-reconstruction over NiFe PBA precursor in chronoamperometry (CA) was applied at constant 1.2 V potential versus Hg/HgO at room temperature using a standard three-electrode setup in 1.0 M KOH electrolytes with and without Fe addition, respectively. All the potentials in linear sweep voltammetry (LSV) was operated for oxygen evolution reaction (OER) performance measurements with a scanning rate of 5 mV s^{-1}. Cyclic voltammetry (CV) was conducted with a potential range of 1.025-1.215 V vs. reversible hydrogen electrode (RHE) at different scan rates without Faradic currents. Operando electrochemical impedance spectroscopy (EIS) tests were obtained at different applied potentials in the frequency range of 0.01-100k Hz. In order to avoid systematic bias and facilitate comparison, all reported potentials for OER performance were converted

against RHE and calibrated with 100% iR compensation by the following equations unless otherwise specified:

$$E_{\text{calibratated}} = E_{\text{measured}} - iR_{\text{s}} \quad \text{(Equation S1)}$$

$$E_{\text{RHE}} = E_{\text{Hg/HgO}} + E^{0}_{\text{Hg/HgO}} + 0.059 \times \text{pH} \quad \text{(Equation S2)}$$

where R_{s} is the series resistance, and $E^{0}_{\text{Hg/HgO}}$ (0.098 V vs. RHE) is the standard potential of Hg/HgO reference electrode at room temperature. The raw data without iR compensation was present in the supplementary Fig. S23. The Tafel slope was obtained by the LSV plots. The electrochemical double-layer capacitances (C_{dl}) were calculated by plotting differences in charging currents ($\Delta j/2$) against v (40, 60, 80, 100, 120 mV s^{-1}) in non-faradic process based on the following equation:

$$C_{\text{dl}} = \frac{\Delta j/2}{v} \quad \text{(Equation S3)}$$

The electrochemically surface area (ECSA) was evaluated by C_{dl} as follows:

$$\text{ECSA} = \frac{C_{\text{dl}}}{C_{\text{s}}} \quad \text{(Equation S4)}$$

where C_{s} is the specific capacitance of planar surface with atomically smooth under identical electrolyte conditions. The value of C_{s} generally is 0.04 mF cm^{-2}.

2. Supplementary Figures and Tables

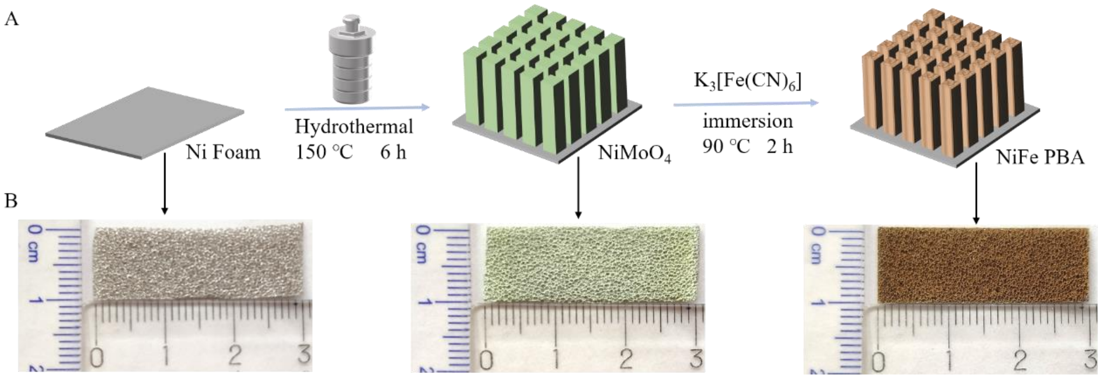

Figure S1. (A) Schematic diagram of synthetic route for NiFe PBA NAs grown on NF. (B) Digital photographs of NF, $NiMoO_4$, and NiFe PBA.

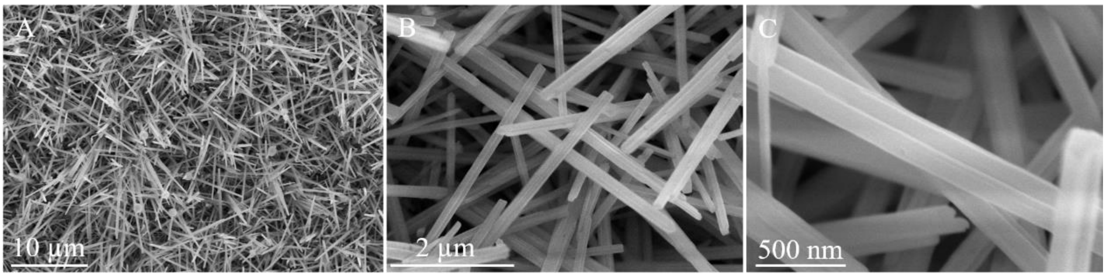

Figure S2. SEM images of $NiMoO_4$ NRs with different scale bars: (A) 10 μm, (B) 2 μm and (C) 1 μm.

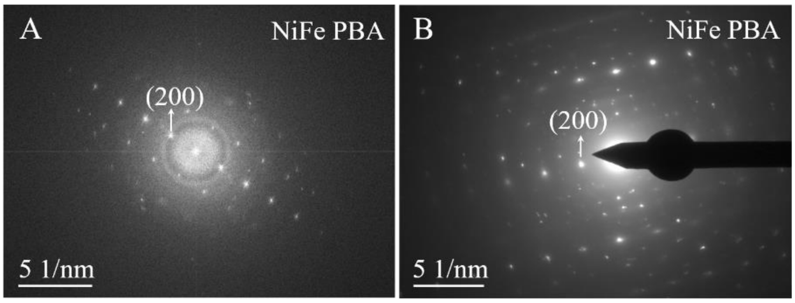

Figure S3. (A) FFT and (B) SAED patterns of NiFe PBA.

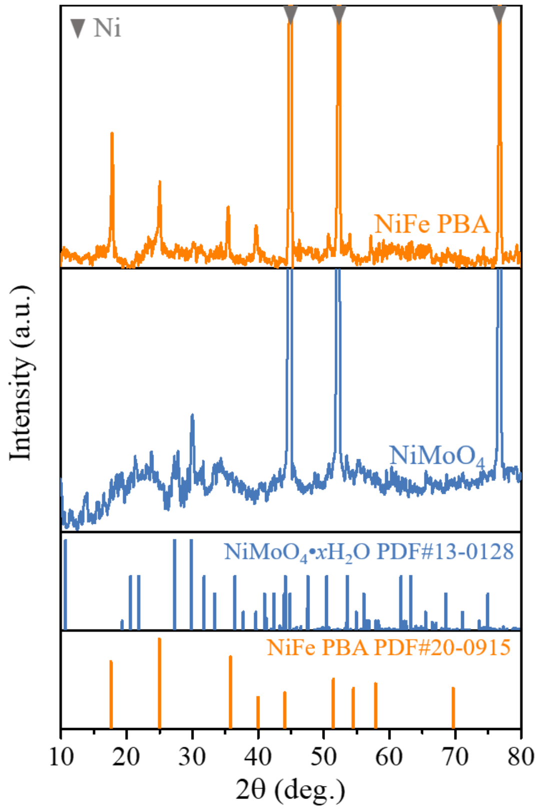

Figure S4. XRD spectra of $NiMoO_4$ and NiFe PBA.

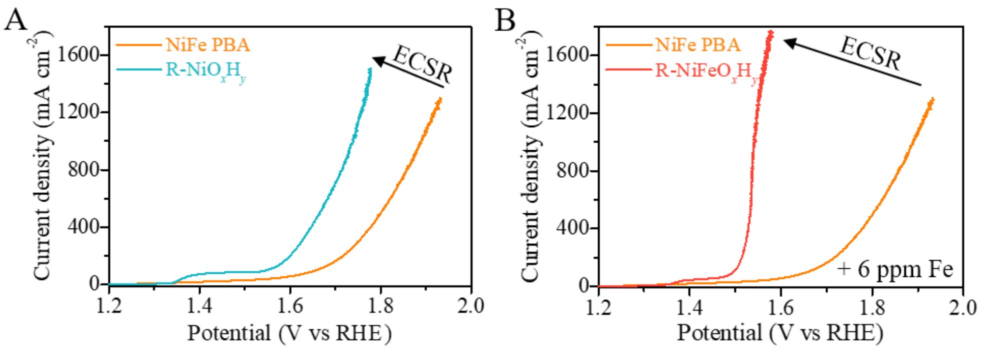

Figure S5. LSV curves of NiFe PBA before and after contrast CA experiments in 1 M KOH electrolytes (A) without and (B) with Fe^{3+} addition.

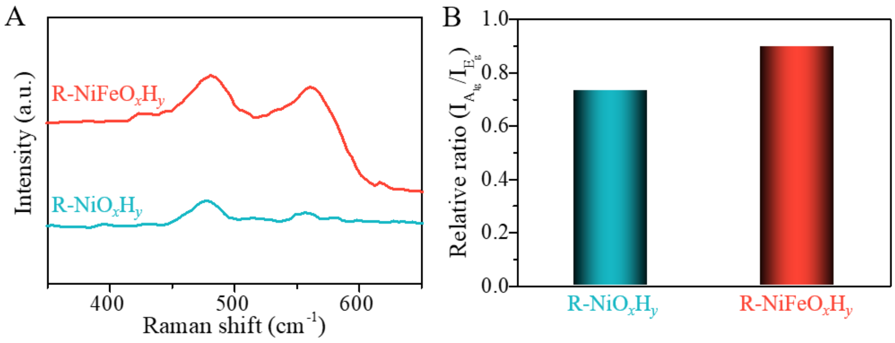

Figure S6. (A) Magnified Raman peaks of Ni^{III}-O bonds and (B) the corresponding $I_{A_{1g}}/I_{E_g}$ ratios for R-NiO_xH_y and R-$NiFeO_xH_y$ products.

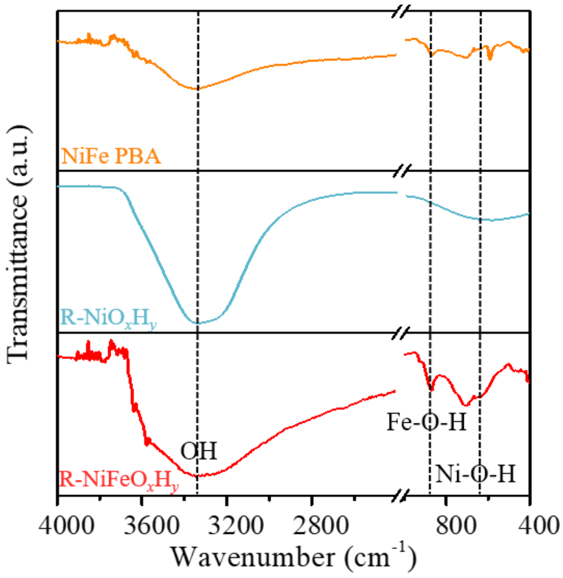

Figure S7. FTIR spectra of NiFe PBA, R-NiO$_x$H$_y$ and R-NiFeO$_x$H$_y$.

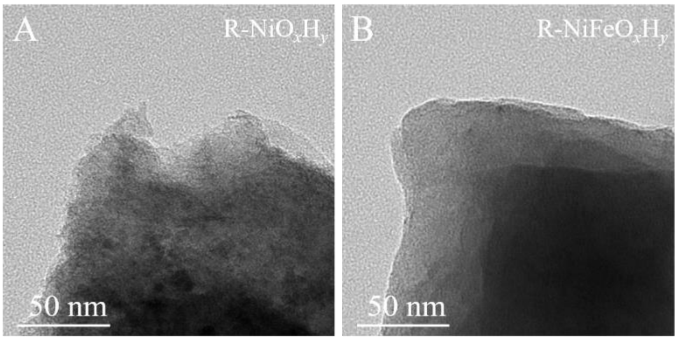

Figure S8. TEM images of R-NiO_xH_y and R-$NiFeO_xH_y$.

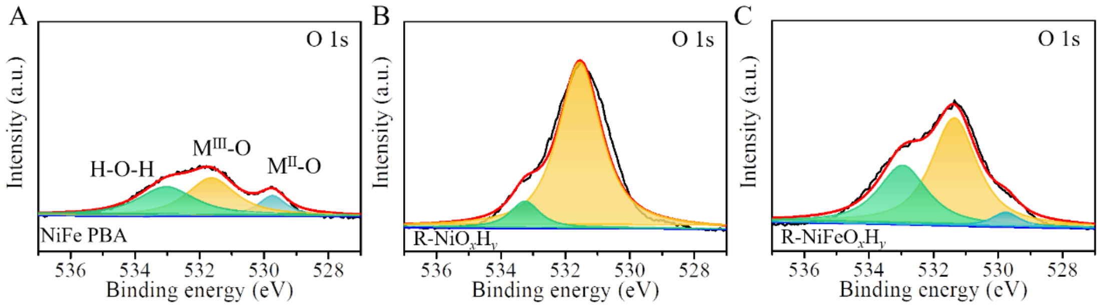

Figure S9. High-resolution O 1s XPS spectra of (A) NiFe PBA, (B) R-NiO_xH_y and (C) R-$NiFeO_xH_y$.

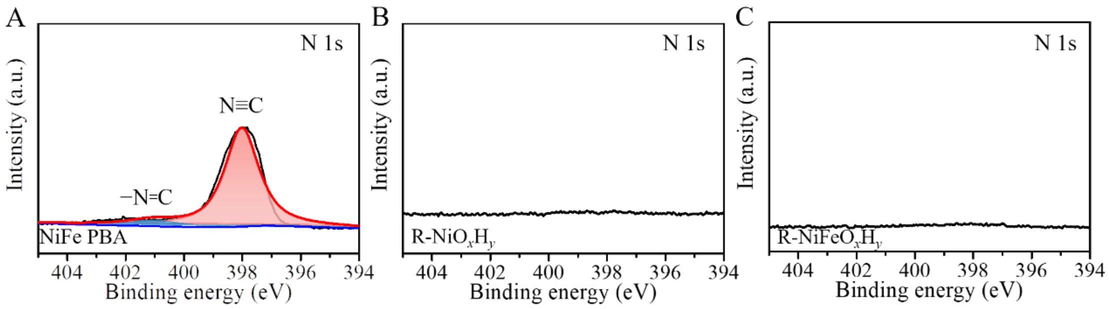

Figure S10. High-resolution N 1s XPS spectra of (A) NiFe PBA, (B) R-NiO$_x$H$_y$ and (C) R-NiFeO$_x$H$_y$.

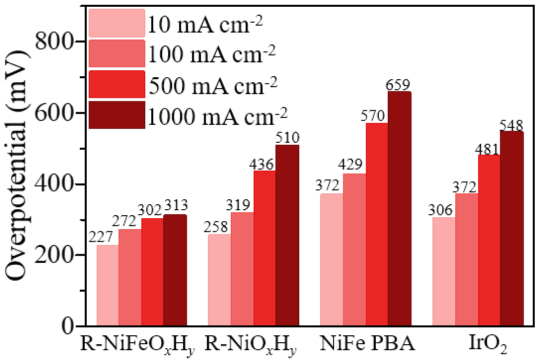

Figure S11. Overpotentials required at 10, 100, 500, 1000 mA cm^{-2} for R-$NiFeO_xH_y$, R-NiO_xH_y, NiFe PBA and IrO_2.

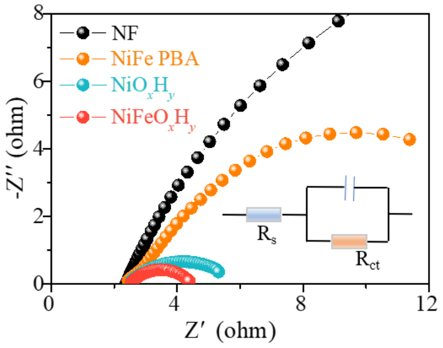

Figure S12. EIS Nyquist plots of R-$NiFeO_xH_y$, R-NiO_xH_y, NiFe PBA and IrO_2.

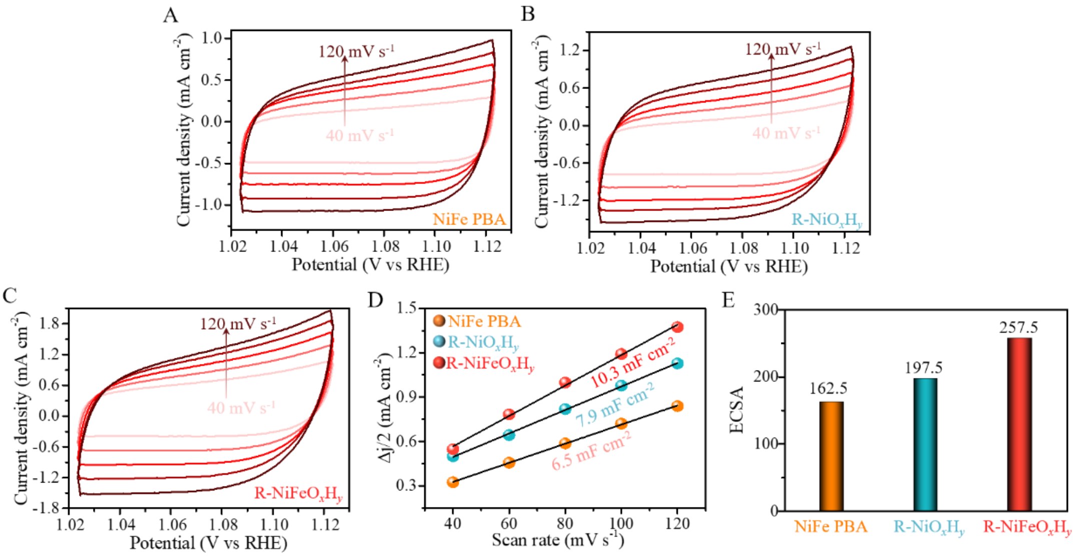

Figure S13. (A-C) CV curves of NiFe PBA, R-NiO_xH_y, and R-$NiFeO_xH_y$ in the non-Faradaic potential region at scan rates from 40 to 120 mV s^{-1}at an interval of 20 mV. (D) DLC and (F) ECSA values of NiFe PBA, R-NiO_xH_y, and R-$NiFeO_xH_y$.

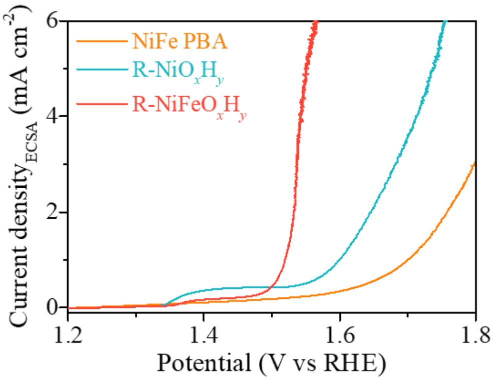

Figure S14. LSV curves normalized by ECSA for NiFe PBA, R-NiO_xH_y, and R-$NiFeO_xH_y$.

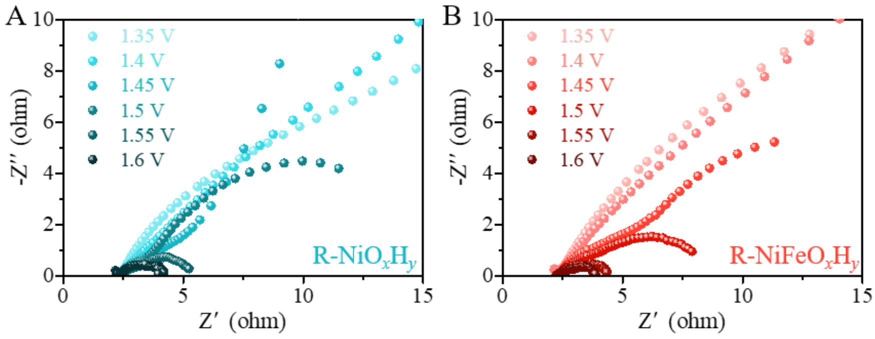

Figure S15. Nyquist plots for (A) R-NiO$_x$H$_y$ and R-NiFeO$_x$H$_y$ at different applied potentials versus RHE in 1 M KOH.

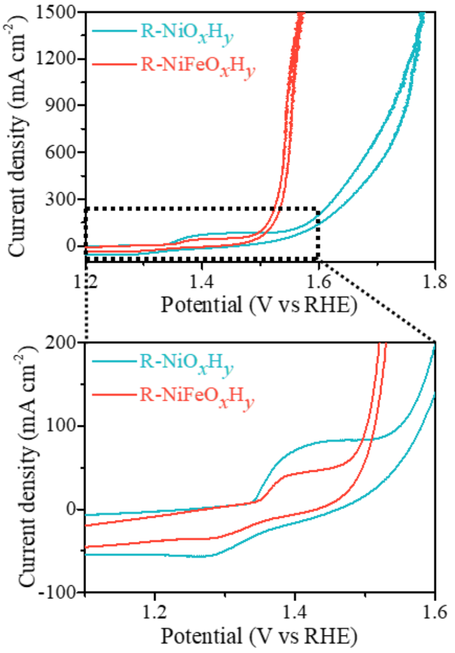

Figure S16. Pseudocapacitive features in CV scanning for R-NiO_xH_y and R-$NiFeO_xH_y$.

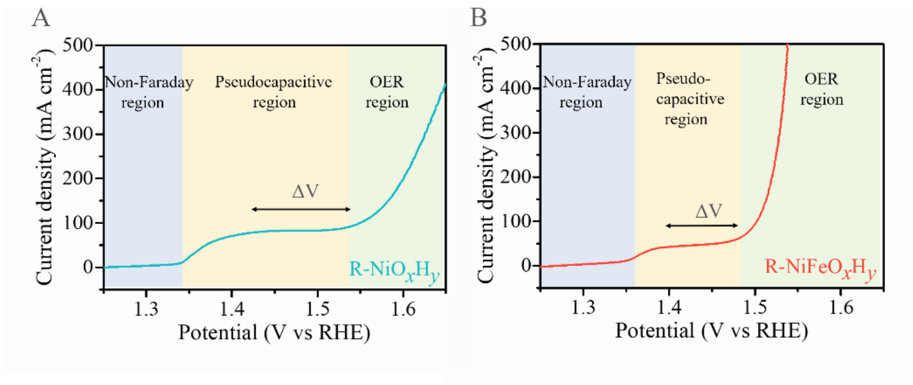

Figure S17. The anodic potential gaps between Ni^{II}/Ni^{III} oxidation and OER onset for (A) R-NiO_xH_y and (B) R-$NiFeO_xH_y$.

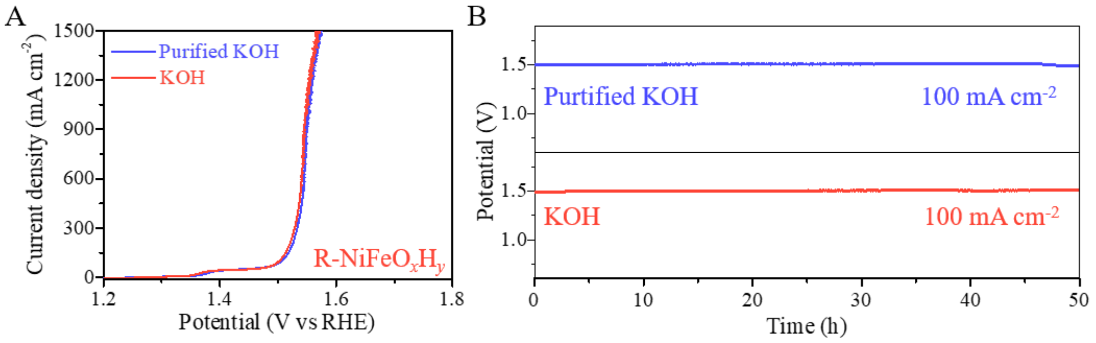

Figure S18. (A) LSV curves, and (B) Chronopotentiometry curves of R-NiFeO$_x$H$_y$ in pristine KOH and purified KOH electrolytes.

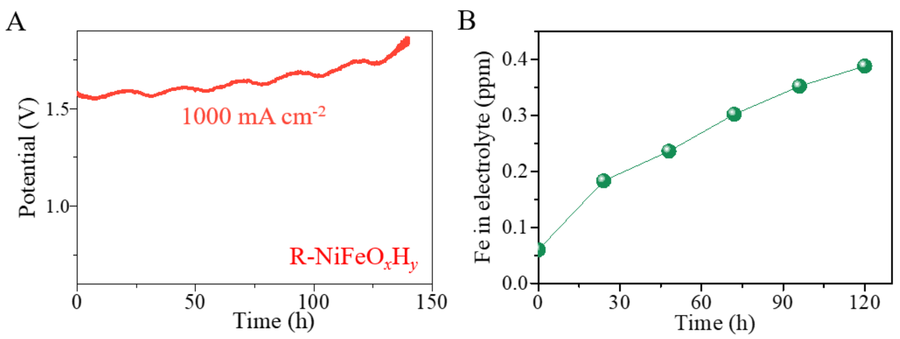

Figure S19. (A) CP test for R-$NiFeO_xH_y$ at 1000 mA cm^{-2}, and (B) the change of Fe ion concentration in electrolyte via in situ ICP-MS measurement.

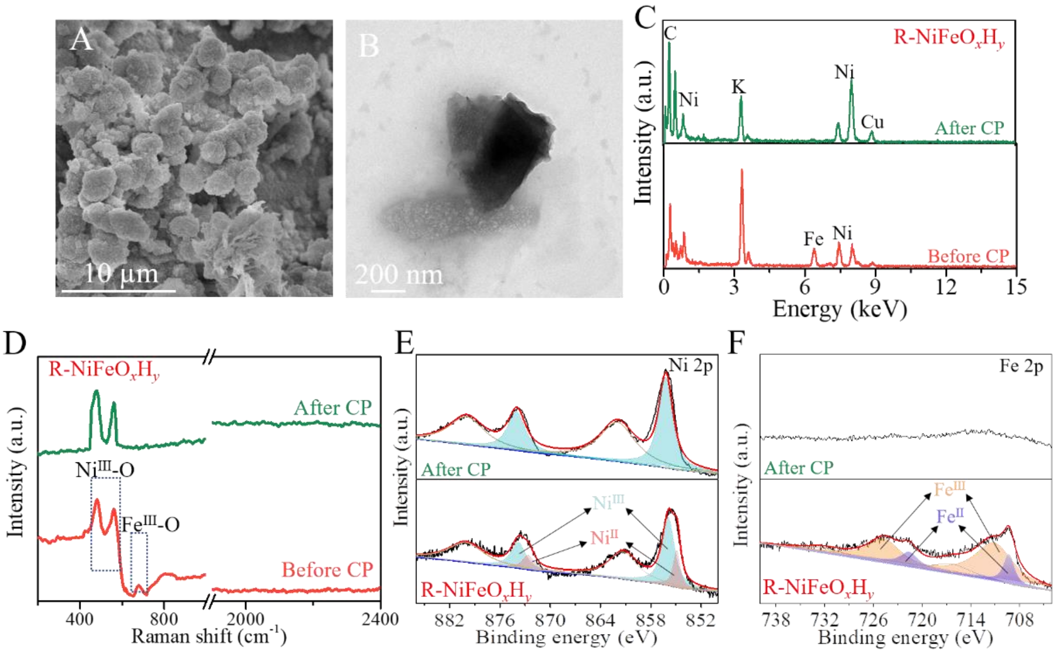

Figure S20. (A) SEM image, (B) TEM image for R-$NiFeO_xH_y$ after CP test at 1000 mA cm^{-2}. (C) EDX spectra, (D) Raman spectra, (E) Ni-2p and (F) Fe-2p XPS spectra for R-$NiFeO_xH_y$ before and after CP test at 1000 mA cm^{-2}.

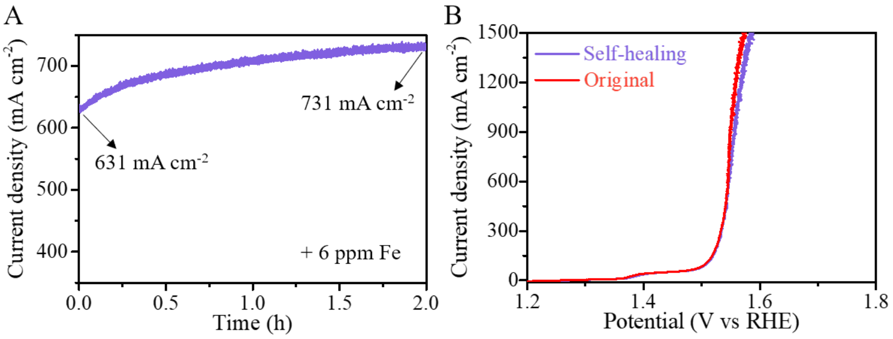

Figure S21. (A) CA retreatment for aged R-$NiFeO_xH_y$ at applied 1.2 V potential in 1 M KOH with 6 ppm Fe addition. (B) LSV curves of original and self-healing R-$NiFeO_xH_y$.

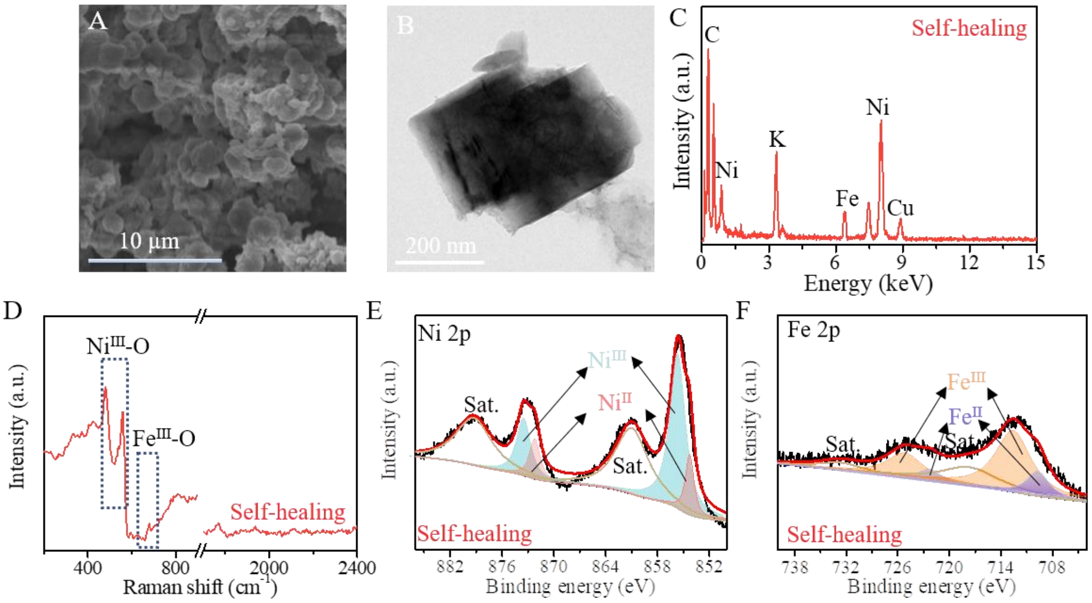

Figure S22. (A) SEM image, (B) TEM image, (C) EDX spectrum, (D) Raman spectrum, (E) Ni-2p and (F) Fe-2p XPS spectra for self-healing R-NiFeO_xH_y.

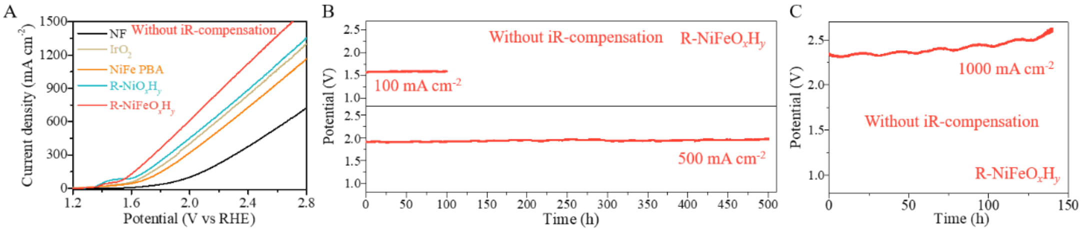

Figure S23. The raw data of (A) LSV and (B-C) CP curves without iR-compensation.

Table S1. ICP-MS data: Cationic Ni and Fe contents in different electrolytes during CA-ECSR processes without and with Fe addition.

Electrolytes	CA Time (h)	ICP ion Concentration (ppm)	
		Ni	Fe
Pristine KOH	0	-0.001	0.060
	1	0.005	1.672
	2	0.009	1.862
	3	-0.073	2.021
	4	-0.047	2.298
	5	-0.092	2.435
	6	-0.098	2.447
+ 6 ppm Fe KOH	0	0.015	6.222
	0.5	0.067	6.359
	1	0.106	6.434
	1.5	0.139	6.495
	2	0.068	6.496

Table S2. XPS data: Cationic Ni and Fe contents in NiFe PBA precursor, R-NiO_xH_y and R-$NiFeO_xH_y$ products.

Catalysts	XPS atomic (%)		Fe survival ratio in catalysts (%)
	Ni	Fe	
NiFe PBA	10.42	5.65	100.00
R-NiO_xH_y	7.36	0.00	0.00
R-$NiFeO_xH_y$	6.11	3.56	63.01

Table S3. XPS data: Cationic Ni and Fe contents with different valence states in NiFe PBA precursor, R-NiO_xH_y and R-$NiFeO_xH_y$ products.

Catalysts	XPS peak area				Ratio of different valence states	
	Ni^{II}	Ni^{III}	Fe^{II}	Fe^{III}	Ni^{II}:Ni^{III}	Fe^{II}:Fe^{III}
NiFe PBA	16129.48	18129.48	9648.57	12148.57	3:2	5:4
	46129.48	27129.48	24148.57	15148.57		
R-NiO_xH_y	0.00	23162.21	0.00	0.00	0:1	0:0
	0.00	49862.21	0.00	0.00		
R-$NiFeO_xH_y$	6504.73	21644.73	6275.68	11975.68	1:3	1:4
	4004.73	9604.73	3175.68	24675.68		

Table S4. A comparison of OER overpotentials at 100, 500 and 1000 mA cm^{-2} for R-$NiFeO_xH_y$ in this work with other advanced catalysts reported in literatures.

Electrocatalyst	Substrate	Electrolyte	J (mA cm^{-2})	Overpotential (mV)	Renferece
Ni_VFe_V LDH	NF	1 M KOH		359	*Angew. Chem. Int. Ed.* **2021**, *133*, 24817
MoNi/NiMoOx@NiFe LDH	NF	1 M KOH		278	*Electrochim. Acta* **2021**, *369*, 137680
S-(Ni,Fe)OOH	NF	1 M KOH	100	300	*Energy Environ. Sci.* **2020**, *13*, 3439
CoS@NiFe LDH	NF	1 M KOH		312	*Small* **2022**, *18*, 2200586
R-$NiFeO_xH_y$	NF	1 M KOH		266	This Work
Ni_xS_y@MnO_xH_y	NF	1 M KOH		356	*Nano-Micro Lett.* **2022**, *14*, 120
Ce-FeOOH	NF	1 M KOH		348	*J. Am. Chem. Soc.* **2019**, *141*, 7005
$(Ni_xFe_yCo_{6-x-y})Mo_6C$	NF	1 M KOH	500	336	*Appl. Catal. B.* **2021**, *290*, 120049
Ni_xFeN/Ni_3N	NF	1 M KOH		288	*J. Mater. Chem. A* **2021**, *9*, 10199
R-$NiFeO_xH_y$	NF	1 M KOH		302	This Work
$FeNiCoCrMnS_2$	NF	1 M KOH		308	*Adv. Funct. Mater.* **2021**, *31*, 2106229
S-(Ni,Fe)OOH	NF	1 M KOH		~355	*Energy Environ. Sci.* **2020**, *13*, 3439
Zn-$Ni_xFe_{1-x}OOH$	NF	1 M KOH	1000	330	*Small* **2022**, *18*, 2203710
P–Ni_3S_2/NiFe	NF	1 M KOH		372	*J. Power Sorces* **2022**, *518*, 230757
R-$NiFeO_xH_y$	NF	1 M KOH		313	This Work

Table S5. A comparison of stability in terms of testing current density and duration time for R-$NiFeO_xH_y$ in this work with other advanced catalysts reported in literatures.

Electrocatalyst	Substrate	Electrolyte	J (mA cm^{-2})	Stability (h)	Renferece
NiFeOOH	CFP	1 M KOH	10	24	*J. Mater. Chem. A* **2021**, *9*, 14043-14051
F-NiFeOOH	NF	1 M KOH		50	*ACS Appl. Mater. Interfaces.* **2021**, *13*, 5142
S-NiFeOOH	NF	1 M KOH		70	*J. Energy Chem.* **2022**, *64*, 364-371
Ov-NiFeOOH	NF	1 M KOH		180	*J. Mater. Chem. A* **2021**, *35*, 20058-20067
NiFe LDH/NiFeOOH	NF	1 M KOH	100	24	*Matter* **2020**, *3*, 1-14
S-(Ni,Fe)OOH	NF	1 M KOH		100	*Energy Environ. Sci.* **2020**, *13*, 3439
NiFeOOH/NiCuOOH	NF	1 M KOH		> 200	*Energy Environ. Sci.* **2020**, *13*, 2200
Ce-NiFeOOH	NFF	1 M KOH		300	*Adv. Funct. Mater.* **2022**, *32*, 2204086
$FeNiCoCrMnS_2$	NF	1 M KOH	500	55	*Adv. Funct. Mater.* **2021**, *31*, 2106229
NiFe LDH	NF	1 M KOH		100	*Appl. Catal. B: Environ.* **2022**, DOI: 10.1016/j.apcatb.2022.122165
NiFeB	RDE	1 M KOH		130	*Nat. Commun.* **2022**, *13*, 6094
R-$NiFeO_xH_y$	NF	1 M KOH		500	This work
